# Chloral in Toothache

**Published:** 1871-11

**Authors:** 


*Chloral in Toothache.*—In the "British Medical Journal," Dr. David Page recommends the introduction into the cavity of a carious tooth of a few grains of the solid chloral hydrate as a remedy in toothache. In a case which he briefly reports, he says, "the toothache was relieved in a few minutes ; aching did not return all night, and, when it did in the morning, was again relieved by the same means."

